# Economic Study of 2-Stage Exchange in Patients With Knee or Hip Prosthetic Joint Infection Managed in a Referral Center in France: Time to Use Innovative(s) Intervention(s) at the Time of Reimplantation to Reduce the Risk of Superinfection

**DOI:** 10.3389/fmed.2021.552669

**Published:** 2021-05-10

**Authors:** Hassan Serrier, Christell Julien, Cécile Batailler, Eugénie Mabrut, Corinne Brochier, Sylvie Thevenon, Marianne Maynard-Muet, Agnes Henry, Sébastien Lustig, Laure Huot, Tristan Ferry, The Lyon BJI Study group

**Affiliations:** ^1^Innovation Unit, Hospices Civils de Lyon, Lyon, France; ^2^Department of Medical Information, Hospices Civils de Lyon, Lyon, France; ^3^Centre Interrégional de Référence Pour la Prise en Charge des Infections Ostéo-Articulaires Complexes (CRIOAc Lyon), Hospices Civils de Lyon, Lyon, France; ^4^Department of Orthopaedic and Sport Surgery, Hôpital de la Croix-Rousse, Hospices Civils de Lyon, Lyon, France; ^5^Université Claude Bernard Lyon 1, Lyon, France; ^6^Clinical Research Centre, Hospices Civils de Lyon, Lyon, France; ^7^Hospital Pharmacy, Hospices Civils de Lyon, Lyon, France; ^8^Infectious Diseases Department, Hospices Civils de Lyon, Lyon, France; ^9^CIRI – Centre International de Recherche en Infectiologie, Inserm, U1111, Université Claude Bernard Lyon 1, CNRS, UMR5308, Ecole Normale Supérieure de Lyon, Univ Lyon, Lyon, France

**Keywords:** prosthetic-joint infection, cost analysis, prevention, antibiotics, cement, healthcare system, superinfection, bone and joint infection

## Abstract

**Objective:** Chronic prosthetic joint infections (PJI) are serious complications in arthroplasty leading to prosthesis exchange and potential significant costs for health systems, especially if a subsequent new infection occurs. This study assessed the cost of chronic PJI managed with 2-stage exchange at the Lyon University Hospital, CRIOAc Lyon reference center, France. A threshold analysis was then undertaken to determine the reimbursement tariff of a hypothetical preventive device usable at the time of reimplantation, which possibly enables health insurance to save money according to the risk reduction of subsequent new infection. This analysis was also performed for a potential innovative device already available on the market, a dual antibiotic loaded bone cement used to fix cemented prosthesis that releases high concentrations of gentamicin and vancomycin locally (G+V cement).

**Method:** Patients >18 years, admitted for a hip or knee chronic PJI managed with 2-stage exchange, between January 1, 2013, and December 31, 2015, were retrospectively identified. Following, resource consumption in relation to inpatient hospital stay, hospitalization at home, rehabilitation care, outpatient antibiotic treatments, imaging, laboratory analysis, and consultations were identified and collected from patient records and taken into account in the evaluation. Costs were assessed from the French health insurance perspective over the 2 years following prosthesis reimplantation.

**Results:** The study included 116 patients (median age 67 y; 47% hip prosthesis). Mean cost of chronic PJI was estimated over the 2 years following prosthesis reimplantation at €21,324 for all patients, and at €51,697 and €15,745 for patients with (*n* = 18) and without (*n* = 98) a subsequent new infection after reimplantation, respectively. According to the threshold analysis the reimbursement tariff (i) should not exceed €2,820 for a device which can reduce the risk of a new infection by 50% and (ii) was between €2,988 and €3,984 if the G + V cement can reduce the risk of a new infection by 80% (this reduction risk is speculative and has to be confirmed by clinical trials).

**Conclusion:** This study revealed that chronic PJI requiring a 2-stage revision is costly, with significant costs in relation to the reimplantation procedure (about 15 k€). However, following reimplantation the rate of subsequent new infection remained high, and the cost of reimplantation following a new infection is considerable, reaching 50k€ per patient. These first cost estimates of managing chronic PJI with 2-stage exchange in France underline the economic interest of preventing new infections.

## Introduction

Infection is the most drastic complication following arthroplasty. In general, the risk of infection is considered to be low (1–2%), but increases by up to 50% in patients with a wide range of cumulative morbidities (1, 2). Debridement and implant retention with mobile part exchange of the prosthesis is the recommended treatment for patients with an acute prosthetic joint infection (PJI) (2, 3). In patients experiencing a relapse following debridement and implant retention or in patients with chronic PJI, a prosthesis revision, i.e., a 1-stage or a 2-stage exchange is recommended, to eradicate the bacteria embedded in biofilm at the surface of the implant. Two-stage exchange is the recommended strategy in the USA and remains a frequent strategy proposed in Europe for knee PJI and for most complex cases, despite more and more surgeons opting to perform 1-stage exchange, especially in France (2–8). PJI is considered to be one of the most costly infectious diseases to treat, as it requires at least one surgery, prolonged hospitalization, rehabilitation care, prolonged antibiotherapy, and extended absence from work in working-age patients. The mean total cost for the management and treatment of septic knee revision in Germany has been calculated to be $12,224 (€11,282) (9), while in the United Kingdom, the mean total costs associated with septic hip revision has been calculated as £21,937 (€24,117) (10). In a study undertaken within a Turkish University Hospital, the median cost of general arthroplasty procedures without PJI (including total hip, total knee, and shoulder) is estimated at $5,937 (€5,479) and increases to $16,999 (€15,689) when PJI occurs (11). When focusing on a two-stage revision, in the Portuguese context, the mean cost of PJI is €11,415 and €13,793 for hips and knees, respectively (12). In contrast, the additional cost associated with the treatment of a hip or knee PJI is estimated at €44,600 for a two-stage revision in Finland (13). Cost also seems to vary considerably depending on the type of pathogen involved, its resistance profile, and if the patient experienced a failure. For instance, in the USA in 2009, the estimated mean cost associated with methicillin-susceptible *Staphylococcus aureus* PJI was $68,053 (€62,823), whereas methicillin-resistant *S. aureus* PJI costs were significantly higher, at a mean of $107,264 (€99,021) per case (14). In Australia, the median cost of treating PJI per patient was AU$34,800 (€19,469), with a 156% increase in case of treatment failure (15). Finally, it is expected that the global cost of PJI will increase in coming decades, especially due to an increase in the absolute number of PJI cases, as the need for joint arthroplasty is expected to increase substantially with population demographic aging. In the USA, the annual cost to hospitals of revision surgery for infection increased from $320 million (€295 million) in 2001 to $566 million (€522 million) in 2009, and was projected to exceed $1.62 billion (€1.49 billion) by 2020 (16).

In this context, it seems essential to prevent septic failures in patients with PJI. These failures are mainly dominated by the onset of a new infection (also called superinfection) that occurs after the reimplantation in 15–30% of the patients for whom a 2-stage exchange was performed (7, 8, 17). To reduce the risk of superinfection, optimization of the classical measures of prevention such as systemic antimicrobial prophylaxis are mandatory at the time of reimplantation (2), and local additional interventions that may further decrease this risk have to be evaluated. In recent years, innovative prevention devices have been developed to prevent PJI. For example, some devices incorporate antibiotics into a bio-absorbable hydrogel or a cement, which can thus be delivered *in situ* (18, 28). Usable during the treatment of PJI or failure, they may increase the probability to avoid certain new infections and therefore reduce the costs of overall treatment. From a payer perspective, these devices could even be profitable, given the high cost of PJI and particularly chronic PJI. In this context, it is important to have high-quality analysis cost data (19) to show the economic impact of PJI, chronic or not, and to estimate costs that could be avoided by using an infection prevention device.

The aim of this study is to assess the cost of knee or hip chronic PJI managed with 2-stage exchange at the CRIOAc Lyon Reference Center. This center belongs to the French CRIOAc network, a nation-wide network with dedicated activity to manage complex bone and joint infection (20). A threshold analysis was then conducted to determine the reimbursement tariff of a hypothetical device usable at the time of reimplantation that would prevent new infection to a point which French health insurance saves money according to the risk reduction. In addition, as the G + V cement is a device already available on the French market and a candidate of interest in such a patient population to fix the cemented prosthesis and potentially contributes to reduce the rate of new infection, the threshold analysis was also performed for this potential innovative device.

## Methods

### Study Characteristics and Data Collection

Patients aged 18 and over, admitted to the CRIOAc Lyon Reference Center for a hip or knee chronic PJI managed with 2-stage exchange, between January 1, 2013, and December 31, 2015, were retrospectively identified. Exhaustivity was checked using the data from the Lyon BJI cohort study. Information about the clinical (infection localization, new infection after reimplantation), demographic (age and gender), and data on resource consumption was collected directly from eligible patients' hospital records. In addition, information on patient care pathway and the outpatient resource consumption which is collected prospectively and recorded in the medical electronic charts as routine care in our institution was included. Information on the management of the osteoarticular infection was also collected, and patients were categorized as follows: explantation then reimplantation (category 1); 1st surgery (usually debridement and implant retention also called DAIR procedure), explantation then reimplantation (category 2); explantation, 2nd look (usually spacer exchange), then reimplantation (category 3); 1st surgery (usually DAIR), explantation, 2nd look (usually iterative DAIR), then reimplantation (category 4). A septic failure was defined in the study as the occurrence, after the reimplantation, of signs of infection (clinical signs of septic arthritis, discharge), leading to the diagnosis of a new episode of PJI (by joint puncture or need for revision). The Ethical Committee of the hospital approved the study (approval No. 17-089); clinical trial number NCT03612076.

### Cost Analysis

A cost study on the 2-stage management of patients with hip or knee PJI at our institution was conducted from the perspective of the French health insurance. Only direct costs, related to the management and treatment of a 2-stage hip or knee procedure, were therefore taken into account and valued using tariffs. Even if the main part of the costs is accumulated during the first year following the reimplantation, a time horizon of 2 years from the reimplantation of the prosthesis was retained in order to take into account the entire impact on resource consumption.

To be exhaustive, our analysis took into account in- and also out-hospital costs including hospital stay, hospitalization at home (HaH), rehabilitation care, outpatient parenteral antimicrobial therapy (OPAT), oral antibiotic treatments, imaging, laboratory analysis, and consultations.

#### Hospital Stay

Data collected from patient files were used to extract information for each patient on all hospital stays from the medico-administrative database of the Hospices Civils de Lyon (program for medicalization of the information systems) during the 2 years following the reimplantation. This method allows us to have exhaustive data on hospital stay and information on the reimbursement tariff of each stay. Only stays related to the management of a hip or knee prosthesis including stays for recurrence and patient follow-up were included in this study. Each stay tariff includes the corresponding diagnosis related group tariff which pays for all the resources consumed during the stay (personnel, implant, laboratory analysis, and imaging) as well as expensive drugs and implantable medical devices that are not included in the diagnosis related group tariff. Of note, hospitalization of patients after the reimplantation is common in France, especially to remove the catheter used for intravenous antimicrobial therapy.

#### Hospitalization at Home and Rehabilitation Care

For HaH and rehabilitation care, the number of days for all stays was available but not the coding used to define the corresponding tariff.

For the HaH, the combination of codes that corresponded to the management of a hip or knee prosthesis was used to define a daily cost. This estimation was then used to value all HaH stays in our study. This association of codes correspond to one of the lowest tariffs possible for HaH, thus a conservative estimation.

For the rehabilitation care, an analysis of the medico-administrative database of two hospitals (Hospices Civils de Lyon and the Val Rosay Hospital) was undertaken corresponding to 311 stays, to estimate a daily cost. This estimation was then used to value all rehabilitation care stays in our study.

The assessment methodology of daily costs in HaH and rehabilitation care is detailed in Supplementary Material 1. These calculation assumptions were also tested in the sensitivity analysis.

#### Out-Hospital Costs

Outpatient oral and/or intravenous antibiotic treatments, consultations, imaging, and laboratory analysis were retained only if they were related to the management of the PJI and if they did not correspond to an episode of in-hospital care, as these costs would be included in the diagnosis related group tariff. Resource consumptions were valued using the current reimbursement tariff of the French health insurance.

Although there were a substantial number of laboratory analyses for each patient in the database, some of them would have only a negligible impact on the overall result (tariff <1€). Moreover, it was also difficult to determine which specific laboratory analyses were related to the disease of interest. Therefore, we chose to focus on the five biological checkups that are most frequently used for the management of hip or knee prosthesis: standard biology including complete blood count or hemogram, blood electrolytes, creatinine, glutamic oxaloacetic transaminase, glutamic pyruvic transaminase, alkaline phosphatase, gamma glutamyl transpeptidase, bilirubine, and c reactive protein; cytochemistry of joint fluid; bacteriological examination; anatomopathological examination; antibiotic dosage. These five biological checkups were included in the analysis only when they were not included in a hospital stay and also valued according to the current reimbursement tariff of the French health insurance.

#### Statistical Analysis

Descriptive analysis was performed on the main characteristics of the population and cost results. A deterministic sensitivity analysis was carried out to test the impact of a modification of the main hypothesis on the result of the evaluation in order to test the uncertainty surrounding these choices.

Pricing information was not available for all stays in a rehabilitation hospital. A mean daily cost of around €255 was estimated based on available data and assigned to all rehabilitation care. The impact of a change of this value to €200 and €300 on the result was tested via the sensitivity analysis.

The discount rate used is 4%; the impact of a modification of this rate to 0% and 6% on our results was also tested.

Finally, to estimate the cost of HaH stays, coefficients corresponding to PCM 04 (post-surgical treatment), ACM 03 or ACM 11 (intravenous treatments or orthopedic rehabilitation), and to a Karnofsky index between 70 and 80% were used. To study the uncertainty around this choice, a more conservative assumption was tested with the same PCM but lowest coefficients for the ACM and the Karnofsky index.

### Threshold Analysis

The objective of this exploratory analysis is to determine the reimbursement tariff per patient for a hypothetical innovative device, usable at the time of reimplantation, below which savings would have been made by the French health insurance if all the patients in our cohort had benefited from the product. However, this evaluation is not realistic since such a device cannot necessarily be used for all patients and will not offer the same effectiveness in all patients.

We have therefore also chosen to carry out this threshold analysis with a concrete example, the use of a cement that releases high concentrations of gentamicin and vancomycin (0.5 g of gentamicin and 2 g of vancomycin per bag of cement powder), referred to here as G+V cement, that has demonstrated *in vitro* its capacity to reduce biofilm formation (18). This device is a bone cement that could be used to fix prosthesis (21). To perform the threshold analysis, we first considered in our cohort, only patients with a cemented prosthesis and removed the cost of the cement used. Then, for patients with a new infection after reimplantation of the prosthesis, the pathogen in question were studied to identify among these infections, those for which the G + V cement could have been active. However, the fact that the product is active does not always mean that the infection would have been avoided. In the lack of clinical studies and *in vivo* data, we hypothesized that G+V cement avoids 80% of the infections for which it is active, based on *in vitro* analysis (22), and based on the wide spectrum of action of this antimicrobial combination (that is potentially active against most of Gram positive and Gram negative pathogens). We thus obtained a number of infections avoided whose cost of care gives us avoided cost attributable to G + V cement in our cohort, based on the drug susceptibility of the pathogens found to be responsible for the superinfection. Assuming that this device had been used for all patients with a cemented prosthesis of our cohort, we can thus estimate the reimbursement tariff of G + V cement below which the French health insurance saves money.

## Results

### Patients' Characteristics

The number of patients included in the study is 116 with a mean age of 66 years old (see Table 1); all patients for whom a 2-stage exchange was performed during the study period were included, except one patient who declined consent. The population is composed of almost as many men (*n* = 57) as women (*n* = 59) and of slightly more patients with a knee (*n* = 61) than a hip prosthesis (*n* = 55). The vast majority (66%) of patients belong to the category 1 of osteoarticular infection management corresponding to “explantation then reimplantation.” During the 2-stage procedure, 71 patients (61%) had a spacer; including 55 patients (77%) for a knee infection, and 16 patients (23%) for a hip infection. Of the 116 patients, 18 patients had a new infection after reimplantation. The main characteristics of the study population are detailed in Table 1. Of note, among the patients with a septic failure, we detailed their management and in particular the surgeries that had been undertaken for these patients in Supplementary Material 2 [median time from reimplantation to failure: 8 weeks (IQR ± 25)]. Concerning the 98 patients without a septic failure, a new surgery was performed in four of them: hip dislocation and revision with a constraint liner at day 20 for one patient; tibial tubercle osteotomy screw removal at month 8 for another patient; patellar resurfacing and soft tissue repair at month 8 for another patient; and 1-stage revision for a mechanical issue at month 18 for the fourth patient.

**Table 1:** Main characteristics of the study population (*n* = 116).

**Characteristics**	***N*** **(%) or mean or median, as appropriate**	
**Age**		
	Mean (SD)	66 (13)
	Median (IQR)	67 (61–74)
**Gender**		
	Female	59 (50.86%)
	Male	57 (49.14%)
**Charlson score**		
	Mean (SD)	3.5
	Median (IQR)	3
**Score ASA**		
	Mean (SD)	2
	Median (IQR)	2
	1	25 (21%)
	2	61 (52%)
	3	29 (25%)
	4	3 (2%)
**BMI**		
	Mean (SD)	29
	Median (IQR)	28
**Type of infection**		
	Monomicrobial infection	76 (66%)
	Polymicrobial infection	21 (18%)
	Undocumented infections	18 (16%)
Type of the initial pathogen^*^		
	*S. aureus*	31 (27%)
	Coagulase-negative Staphylococci	26 (22%)
	*Streptococcus spp*.	13 (11%)
	*Cutibacterium acnes*	15 (13%)
	*Enterococcus faecalis*	5 (4%)
	*Corynebacterium spp*.	4 (3%)
	*Pseudomonas spp*.	1 (1%)
	*Burkholderia spp*.	1 (1%)
	*Actinomyces neurrii*	1 (1%)
	*Veillonela spp*.	1 (1%)
**Osteoarticular infection categories**		
	Category 1	76 (65.52%)
	Category 2	21 (18.1%)
	Category 3	13 (11.21%)
	Category 4	6 (5.17%)
**Infection localization**		
	Hip	55 (47.41%)
	Knee	61 (52.59%)

* *at the time of explantation or at the time of DAIR before explantation*.

*SD, standard deviation; IQR, interquartile range; ASA, American society of anesthesiologists; BMI, body mass index; DAIR, debridement antibiotics and implant retention*.

*Osteoarticular categories: explantation, then reimplantation (category 1); 1st surgery (usually debridement and implant retention also called DAIR procedure), explantation, then reimplantation (category 2); explantation, 2nd look (usually spacer exchange), then reimplantation (category 3), 1st surgery (usually DAIR), explantation, 2nd look (usually iterative DAIR), then reimplantation (category 4)*.

### Cost Analysis

The mean cost of 2-stage management care of patients with knee or hip PJI at the Hospices Civils de Lyon is estimated at €21,324 over 2 years from the reimplantation of the prosthesis (see Table 2). Hospital stays and rehabilitation care are the two main cost items. They represent, respectively, 34 and 61.52% of the total cost. Cost of antibiotics is very low because it only concerns antibiotics not included in a hospital stay. Most of them are delivered at the hospital and consequently included in the tariff of the stay.

**Table 2:** Cost of care by follow-up year and type of resource consumption per patient.

	**Year 1**		**Year 2 (discounted)**		**Two years cost per patient in €**	
	**Mean cost in €(SD)**	**%**	**Mean cost in €(SD)**	**%**	**Mean cost in €(SD)**	**%**
Hospital stays	5,173 (8,988)	71.35	2,077 (6,799)	28.65	7,250 (12,713)	34
HaH	431 (1,949)	73.02	159 (1,714)	26.98	590 (2,569)	2.77
Rehabilitation care	11,313 (18,883)	86.24	1,805 (12,164)	13.76	13,118 (26,637)	61.52
**Out-hospital costs**						
Antibiotics	16 (45)	80.29	4 (24)	19.71	20 (50)	0.09
Consultations	61 (45)	75.65	20 (28)	24.35	81 (64)	0.38
Biology	165 (229)	81.57	37 (100)	18.43	203 (252)	0.95
Imagery	48 (70)	76.28	15 (29)	23.72	62 (84)	0.29
Total	17,207 (24,661)	80.69	4,117 (15,270)	19.31	21,324 (33,457)	100

There was at least one hospitalization for 75 patients in the first year of follow-up and for 21 patients in the second year. Only 35 patients had no hospitalization during the 2 years of follow-up. The average duration of a new hospitalization is ~8 days with a median of 5 days.

Only 11 patients had no stay in a rehabilitation hospital during the first year compared with 111 in the second year. The average length of stay in a rehabilitation hospital is 66 days with a median of 42 days.

The main part (80.69%) of the costs is accumulated in the first year following the reimplantation of the prosthesis (see Table 2). Mean cost of patient care is estimated at €17,207 for the first year and €4,117 euros for the second year.

Expensive drugs and implantable medical devices not included in diagnosis related group only represent 1.09% and 3.61% of the mean cost of hospital stays, respectively.

In terms of gender, the average cost of care is estimated at €22,932 for females and €19,660 for males (see Table 3).

**Table 3:** Subgroup analysis of costs.

			**Two years cost in €**		
		***N***	**Mean (SD)**	**Median (IQR)**	**Min-Max**
Total		116	21,324 (33,457)	11,677 (5,033–24,325)	743–253,742
**Gender**					
	Female	59	22,932 (31,492)	14,307 (8,559–24,484)	743–204,917
	Male	57	19,660 (35,580)	8,957 (4,701–16,783)	897–253,742
**Osteoarticular infection**					
	Category 1	76	18,329 (19,005)	12,269 (5,938–24,325)	743–117,977
	Category 2	21	22,506 (55,524)	3,966 (2,556–13,363)	1,838–253,742
	Category 3	13	24,792 (19,149)	21,131 (10,678–36,342)	1,078–64,438
	Category 4	6	47,611 (78,184)	109,56 (8,502–34,977)	8,167–204,917
**Infection localization**					
	Hip	55	22,152 (32,516)	14,208 (5,782–23,269)	897–204,917
	Knee	61	20,577 (34,535)	10,678 (4,981–24,802)	743–253,742
**New infection after reimplantation**					
	Without	98	15,745 (18,144)	10,369 (4,957–17,756)	753–117,977
	With	18	51,697 (67,361)	36,623 (18,050–45,025)	1,078–253,742

*Osteoarticular categories: explantation, then reimplantation (category 1); 1st surgery (usually debridement and implant retention also called DAIR procedure), explantation, then reimplantation (category 2); explantation, 2nd look (usually spacer exchange), then reimplantation (category 3), 1st surgery (usually DAIR), explantation, 2nd look (usually iterative DAIR), then reimplantation (category 4)*.

Among the categories of osteoarticular infections management, category 4 corresponding to a 1st surgery, 2nd look, explantation, then reimplantation is the most expensive with a mean cost estimated at €47,611 per patient (see Table 3). The mean cost for category 1 patients (explantation, then a reimplantation), estimated at €18,329 per patient, is the lowest.

Mean costs are relatively close for patients with a knee infection and for those with a hip infection.

The mean cost is estimated at €51,697 for patients with a new infection after reimplantation and at €15,745 for patients without.

### Threshold Analysis

Considering that all 116 patients of our cohort could have benefited from an innovative device that may prevent new infection, we estimated, according to the number of new infections avoided, the reimbursement tariff below which the French health insurance saves money (see Table 4). For example, if this innovative device avoids 50% of infections, nine patients would have had no infection. Therefore, the French health insurance could save money for a reimbursement tariff below €2,820 per patient.

**Table 4:** Reimbursement tariff of the preventive innovative device per patient in € below which health insurance saves money depending on the number of avoided infections.

**Numbers of patients with avoided infection**	**1**	**2**	**3**	**4**	**5**	**6**	**7**	**8**	**9**
Threshold per patient in €	313	627	940	1,253	1,567	1,880	2,194	2,507	2,820
**Numbers of patients with avoided infection**	**10**	**11**	**12**	**13**	**14**	**15**	**16**	**17**	**18**
Threshold per patient in €	3,134	3,447	3,760	4,074	4,387	4,701	5,014	5,327	5,641

Of the 18 patients with the new infection after reimplantation, based on the antibiogram, the G + V cement could have been active for 12 (see Table 5); however, only nine had a cemented prosthesis (3 hip and 6 knee prosthesis). In the hypothesis that G + V cement avoids 80% of the infections for which it is active, we estimate that the infection could have been avoided for 6 to 8 patients.

**Table 5:** Description of the pathogens and their susceptibility to gentamicin + vancomycin combination for the 18 patients with a failure after prosthesis reimplantation.

**ID patient**	**Pathogen identified**	S^*^ to G + V	**Pathogen identified**	S^*^ to G + V	**Pathogen identified**	S^*^ to G + V	**Pathogen identified**	S^*^ to G + V	**Pathogen identified**	S^*^ to G + V	**Conclusion about the potential activity of the G + V cement**
1	*P. aeruginosa*	No									No
2	Streptococcus^**^	Yes									Yes
3	Without documentation	UNK									UNK
4	MRSE	Yes									Yes
5	*E. aerogenes*	Yes	*P. aeruginosa*	Yes							Yes
6	*MDR E. cloacae*	Yes	*E. faecalis*	Yes							Yes
7	*MDR K. pneumoniae*	No									No
8	MRSE	Yes									Yes
9	*C. albicans*	No	*K. pneumoniae*	Yes	*C. tuberculostearicum*	Yes					No
10	*E. cloacae*	No	*E. faecium*	Yes							No
11	MRSE	Yes									Yes
12	MSSA	Yes	MSSE	Yes							Yes
13	*S. agalactiae*	Yes									Yes
14	MSSA	Yes									Yes
15	*S. lugdunensis*	Yes									Yes
16	MRSE	Yes									Yes
17	MSSA	Yes	*S. agalactiae*	Yes	*F. magna*	yes					Yes
18	MSSA	Yes	*B. fragilis*	No	*C. koserii*	Yes	*P. mirabilis*	No	*F. magna*	Yes	No

*MRSE, methicillin-resistant S. epidermidis; MDR, multidrug-resistant; MSSA, methicillin-susceptible S. aureus; MSSE, methicillin-susceptible S. epidermidis;*

* *Susceptible to the combination gentamicin plus vancomycin;*

** *obtained by PCR*.

By removing the cost of the cement, the average cost of care is €52,020 for patients with infection after reimplantation against €15,669 for patients without. The cost that could have been avoided if the infection had been avoided is therefore estimated at €36,351 per patient.

According to these assumptions, the G + V cement cost per patient below which the avoided costs are higher than the extra costs is estimated between €2,988 and €3,984 (see Table 6).

**Table 6:** G + V cement cost threshold per patient in € below which health insurance saves money depending on the number of avoided infections.

**Numbers of patients with avoided infection**	**1**	**2**	**3**	**4**	**5**	**6**	**7**	**8**	**9**
Threshold per patient in €	498	996	1,494	1,992	2,490	2,988	3,486	3,984	4,482

### Deterministic Sensitivity Analysis

**Figure 1:**
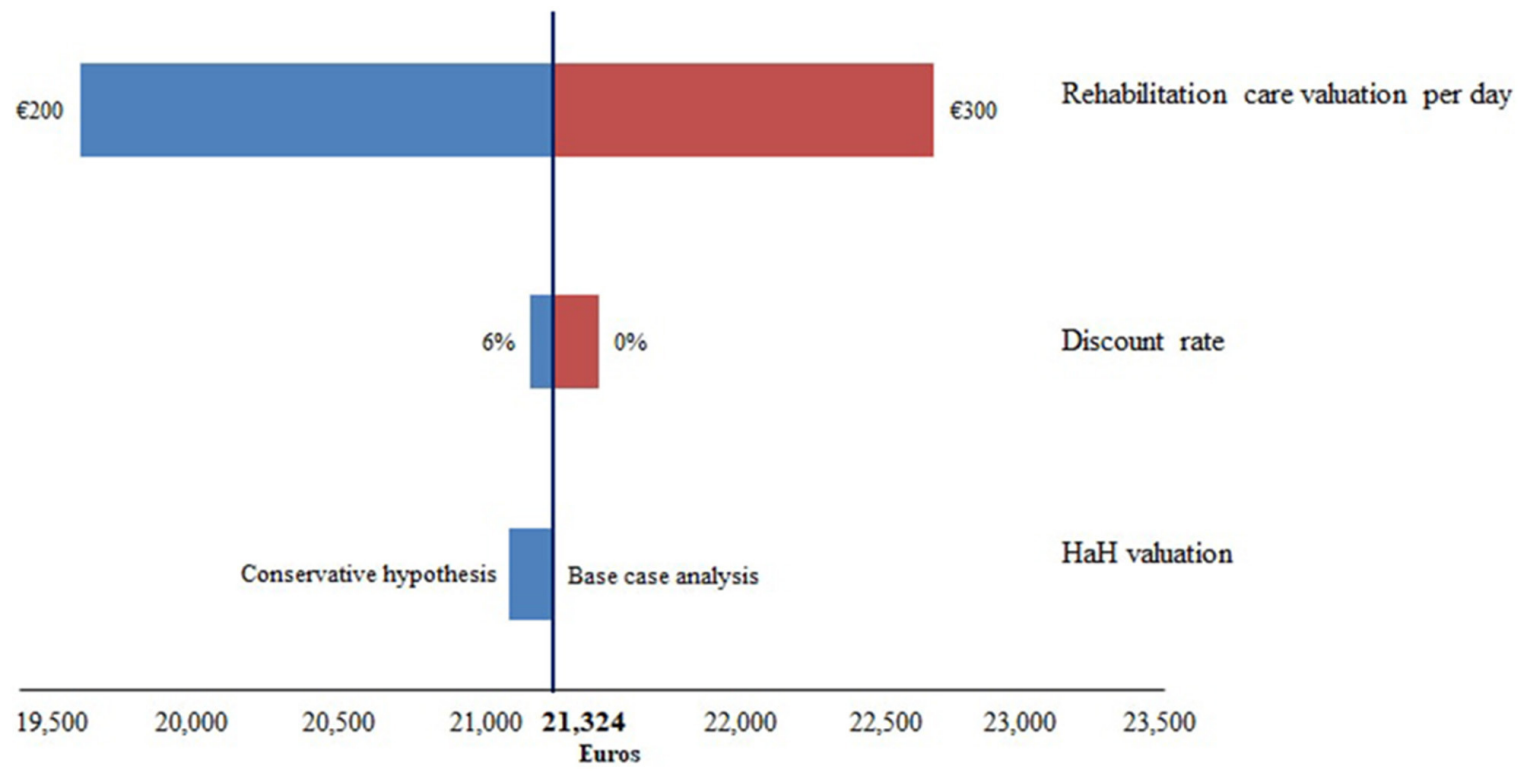
Tornado diagram on the impact of methodological choices on the mean cost of care per patient.

The discount rate has a low impact on the mean cost of patient care. If the rate varies from 6 to 0% the mean cost increases from €21,246 euros to €21,488 (see Figure 1).

The choice concerning the HaH valuation also has little impact on the results. The transition to a conservative hypothesis reduces the average cost of €157.

The choice that impacts the most the results is the valuation of rehabilitation care. For daily costs of rehabilitation care of €200 and of €300, the mean costs per patient are estimated, respectively, at €19,635 and €22,688.

## Discussion and Conclusion

The mean cost of 2-stage management care of patients with knee or hip PJI at the CRIOAc Lyon was estimated at €21,324 over 2 years from the reimplantation of the prosthesis. Through the exhaustive collection of all in- and out-hospital resource consumption, we demonstrate that the main component of the costs is accumulated in the first year following the reimplantation of the prosthesis. Hospital stays and rehabilitation care are by far the two main cost items. We find that the mean cost of management care is estimated at €51,697 for patients with a subsequent new infection after reimplantation and at €15,745 for patients without infection after reimplantation. Even if the sample size of patients with a new infection is small (*N* = 18), the difference in the cost of treating a patient with a new infection compared to a patient without a new infection is substantial (€35,952). Beyond the individual consequences for the patient that includes a potential loss of function due to the management of a septic relapse, this cost is considerable for the French health care system.

Our study is the only one to assess the cost of PJIs in the French context from a health insurance perspective. Our results are not comparable with those of the literature. Without being exhaustive, we have, however, attempted to discuss the consistency of our result by comparing them first with available French studies and then foreign studies whose objective and methodology are closest to this study.

A study previously evaluated the cost of hip PJI in France (23), but this study was conducted from the perspective of the hospital and not of the payer as in this study. Costs are therefore valued using the production cost when possible and not the tariff. The mean cost of hip PJI was thus estimated at €32,546 against €22,152 in our study (including patient with and without new infection after reimplantation). We estimated the mean cost of hip or knee PJI at €15,745 for patients without new infection after reimplantation. The mean total costs for septic revision were £21,937 (€24,117) for total hip replacements in United Kingdom (10) and $12,224 (€11,282) for total knee arthroplasty in Germany (9). The median cost of arthroplasty (including total hip, total knee, but also shoulder) was estimated at $16,999 (€15,689) with PJI in a Turkish University hospital (11). Focusing on a two-stage revision, in the Portuguese context the mean cost of PJI was €11,415 and €13,793 for, respectively, hips and knees (12). Despite the differences in term of context and perspective, our mean hip or knee PJI cost estimate seems to be consistent with the literature.

We estimated the excess mean cost at €51,697 for hip or knee patients with a new infection after reimplantation. In the study of Puhto et al. (13), 8 patients who failed on debridement, antibiotics, and implant retention were treated in a two-stage revision. The excess cost of a hip or knee PJI was estimated at €44,600 for these patients in Finland. Peel et al. (15) estimated the mean cost of hip or knee PJI with a failure at AU$66,426 (€36,462). The costs are evaluated from the payer perspective, over a period of 3 years, but does not take into account rehabilitation care costs, which our study found to be a significant consumption of resources. The use of rehabilitation care is undoubtedly more important in the event of a new infection, which could partly explain this difference.

Furthermore, the sensitivity analysis shows that the valuation of rehabilitation care has the greatest impact on the mean cost of care. However, the value we retained is robust since it is based on the data of 311 stays from administrative databases of two hospitals (Hospices Civils de Lyon and at the Val Rosay hospital). The uncertainty, relative to our assumptions, which surrounds our results is therefore relatively low.

One of the main limitations of our results is the low number of patients with a new infection after reimplantation, which makes the comparison with patients without a new infection statistically fragile. Only a bootstrap would have enabled statistically robust comparisons to be made but our sample of patients with a new infection is also too low to perform resampling in a robust way. However, the difference between the two groups is substantial in terms of cost (€35,952) and could probably be confirmed in a future study on a larger sample.

In line with the perspective retained in our analysis, that of the French health insurance, we do not take into account the loss of patient productivity or the costs of informal care provided to the patient. Our estimates therefore do not reflect the societal burden of PJI, which is undoubtedly much greater.

As a consequence, prevention of infection is crucial in patients managed for a PJI. As our Reference Center has already set up all the recommended prophylaxis guidelines by using a checklist that includes WHO SSI prevention recommendations (24), innovative approaches by using particular devices that have the ability to act locally to reduce the rate of post-operative infection is now required for such patients. New generations of cement that release a combination of high doses of antimicrobials are candidates for that purpose. Based on clinical data from arthroplasty registers published in the early 2000s, systemic antibiotics combined with gentamicin loaded cement in patients for whom a cemented prosthesis is required are considered to be the most effective prophylaxis against deep infection (25). Recently, high dose dual antibiotic impregnated cements have been developed, such as a cement that releases a combination of high concentrations of gentamicin and clindamycin antibiotics. A quasi-randomized study showed that the rate of infection was lower when using this cement in comparison with standard low dose gentamicin cement in patients for whom hemiarthroplasty was performed following hip fracture, a patient group generally susceptible to PJI (26). In patients with PJI managed by a 2-stage approach, the rate of clindamycin-resistant and multidrug-resistant pathogens is particularly high and the spectrum of activity of the combination of gentamicin plus vancomycin seems to be more appropriate. *In vitro*, recent results suggest that the G + V cement, which is a bone cement available on the market that could be used to fix prosthesis and release a combination of high concentrations of gentamicin and vancomycin antibiotics, increases the anti-biofilm prophylactic effect compared to cement loaded with gentamicin alone (22). These findings were especially relevant for clinical strains of *S. aureus* and gentamicin-resistant staphylococci. A gentamicin + clindamycin (G + C) cement was also tested *in vitro* in this study and of note, G + C cements are also available in the market. The tested G + C cement has also anti-biofilm prophylactic effect *in vitro*, but clindamycin resistance is much more common than vancomycin resistance (27). As a consequence, the spectrum of activity of G + V cements seems to be more interesting, even if the dose of gentamicin, in the tested G + C cement, is higher in comparison with that of the G + V cement. Here, based on the antibiogram of the pathogen responsible for the new infection in patients managed with a 2-stage approach, there would be an added value in using the G + V cement. As a consequence, this cement is of interest in such a population, to fix the cemented prosthesis and to potentially contribute to reduce the rate of new infection.

The threshold analyses performed here are of importance as depending on the preventive efficacy of the intervention, the money saved could be calculated. For instance, if all patients within our cohort had benefited from an innovative device that prevents 50% of new infection, the French health insurance would have saved money for a reimbursement tariff at or below €2,820 per patient, while G + V cement is cost effective if it is less than between €2,988 and €3,984 per patient, depending on the hypothesis based on the number of new infections avoided. Therefore, the additional cost related to the use of G + V cement in the population studied would have been offset by the new infections avoided and their associated costs.

This study represents to date the only assessment of the cost of chronic hip or knee PJI in France, but it has some limitations. Comparisons of our results with the literature are somewhat complicated because the methodologies (perspective, time horizon, costs taken into account, etc.) and health systems are different. Although the sample size is small and even if our study is monocentric, the data presented here are the first cost estimates of 2-stage management care of patients with knee or hip chronic PJI in France, and underline the economic interest of preventing new infections after reimplantation. Finally, clinical studies are crucial to confirm the measurable efficacy of a device of interest, and the proposed calculator will be a valuable tool to set the correct price for such a medical device in this specific application.

To conclude, this study revealed that chronic PJI requiring a 2-stage revision is a costly indication, with a significant cost of the reimplantation procedure alone (~15 k€ in patients without a new infection). However, the rate of new infection continues to remain high, and the additional cost of reimplantation following a new infection is considerable, reaching ~50 k€ per patient. These first cost estimates of knee or hip chronic PJI managed in 2-stage exchange in France underline the economic interest of preventing subsequent new infections, especially by using cost effective innovative devices that need to be evaluated in prospective studies.

## Data Availability Statement

The raw data supporting the conclusions of this article will be made available by the authors, without undue reservation.

## Ethics Statement

The studies involving human participants were reviewed and approved by Hospices Civils de Lyon ethic committee. Written informed consent from the participants' legal guardian/next of kin was not required to participate in this study in accordance with the national legislation and the institutional requirements.

## Author Contributions

HS, CJ, EM, CBa, ST, MM-M, LH, and TF worked on the study conception and design. CJ, EM, CBr, ST, MM-M, and TF contributed to acquire data. HS analyzed data. HS and TF have been involved in drafting the manuscript. CBa, AH, and SL revised critically the manuscript. All authors read and approved the manuscript final version to be published.

### Lyon Bone and Joint Study Group (List of Collaborators)

**Coordinator:** Tristan Ferry; **Infectious Diseases Specialists**—Tristan Ferry, Florent Valour, Thomas Perpoint, Patrick Miailhes, Florence Ader, Sandrine Roux, Agathe Becker, Claire Triffault-Fillit, Anne Conrad, Cécile Pouderoux, Nicolas Benech, Pierre Chauvelot, Marielle Perry, Fatiha Daoud, Johanna Lippman, Evelyne Braun, and Christian Chidiac; **Surgeons**—Sébastien Lustig, Elvire Servien, Cécile Batailler, Stanislas Gunst, Axel Schimdt, Matthieu Malatray, Eliott Sappey-Marinier, Michel-Henry Fessy, Anthony Viste, Jean-Luc Besse, Philippe Chaudier, Lucie Louboutin, Quentin Ode, Adrien Van Haecke, Marcelle Mercier, Vincent Belgaid, Arnaud Walch, Sébastien Martres, Franck Trouillet, Cédric Barrey, Ali Mojallal, Sophie Brosset, Camille Hanriat, Hélène Person, Nicolas Sigaux, Philippe Céruse, and Carine Fuchsmann; **Anesthesiologists**—Frédéric Aubrun, Mikhail Dziadzko, and Caroline Macabéo; **Microbiologists**—Frederic Laurent, Laetitia Beraut, Tiphaine Roussel-Gaillard, Céline Dupieux, Camille Kolenda, and Jérôme Josse; **Pathologist**—Alexis Trecourt; **Imaging**—Fabien Craighero, Loic Boussel, Jean-Baptiste Pialat, and Isabelle Morelec; **PK/PD specialists**—Michel Tod, Marie-Claude Gagnieu, and Sylvain Goutelle; **Clinical research assistant and database manager**—Eugénie Mabrut.

## Conflict of Interest

TF received a speaker honorarium from Heraeus Medical GmbH in 2017 [Symposium EBJIS 2017; Rationale for the use of local antibioticS in patients with septic pseudarthrosis (septic non-union)]. The remaining authors declare that the research was conducted in the absence of any commercial or financial relationships that could be construed as a potential conflict of interest.

## Abbreviations

PJI: Prosthetic Joint Infection
HaH: hospitalization at home
PCM: primary care mode
ACM: associated care mode.
