# Supplementary material for: Economic Study of 2-Stage Exchange in Patients With Knee or Hip Prosthetic Joint Infection Managed in a Referral Center in France: Time to Use Innovative(s) Intervention(s) at the Time of Reimplantation to Reduce the Risk of Superinfection
Source: Front Med (Lausanne). 2021 May 10;8:552669. doi: 10.3389/fmed.2021.552669 (PMC8142816; doi:10.3389/fmed.2021.552669)
Supplement: Supplementary file 1 [file Data_Sheet_1.docx]

Supplementary Material S.1 Detailed cost assessment methodology.

A cost study on the 2-stage management of patients with hip or knee PJI at our institution was conducted from the perspective of the French health insurance. Only direct costs, related to the management of a hip or knee prosthesis, were therefore taken into account and valued using tariffs. Even if the main part of the cost is accumulated in the first year following the reimplantation, a time horizon of two years from the reimplantation of the prosthesis was evaluated in order to take into account the entire impact on resource consumption.

In order to be exhaustive, our analysis took into account in- and also out-hospital costs including hospital stay, hospitalization at home (HaH), rehabilitation care, outpatient parenteral antimicrobial therapy (OPAT), oral antibiotic treatments, imaging, biology acts, and consultations.

Costs occurring the second year following the reimplantation were discounted according to guidelines using a discount rate of 4 % ([HAS, 2012](#_ENREF_9); [Drummond et al., 2015](#_ENREF_4)). We tested the impact of a modification of the value of this rate in a sensitivity analysis.

**Hospital stay**

Data collected from patient files were used to extract information for each patient on all hospital stays from the medico-administrative database of the Hospices Civils de Lyon (program for medicalization of the information systems) during the two years following the reimplantation. This method allows us to have exhaustive data on hospital stay and information on the reimbursement tariff of each stay. Only stays related to the management of a hip or knee prosthesis including stays for recurrence and patient follow-up were included in this study. Each stay tariff includes the corresponding diagnosis related group tariff which pays for all the resources consumed during the stay (personnel, implant, biology acts and imagingetc.) as well as expensive drugs and implantable medical devices that are not included in the diagnosis related group tariff.

**Hospitalization at Home**

HaH is a particular care modality that allows complex care of patients (in theory, the same as that provided in the hospital) at home. Medico-administrative data like those available for in-hospital stays were not available for HaH. Therefore, the number of days of HaH were calculated for each patient based on the information available in their patient records.

The care of patient in HaH is characterized by a combination of a primary care mode (PCM),if applicable, an associated care mode (ACM), and a value of the Karnofsky index. This index described, on a generic scale from 0 (meaning death) to 100% (meaning no sign or symptom of illness), the patient's overall state of health, the help the patient requires for the gestures of life (personal needs, dressing, etc.) and the medical care required (Karnofsky, 1949). The multiplication of the weights corresponding to each of these elements (PCM, ACM and Karnofsky index) with the coefficients corresponding to each period of the stay (1st to 4th day / 5th to 9th day / 10th to 30th day / from 31st day) allows us to obtain a total weighting index for each period. The value of the total weighting index determines the homogeneous group of the tariff. There only 31 homogeneous groups of tariffs for HaH.

No information was available on the coding of stays in HaH. The most relevant association of PCM, ACM and Karnofsky index and valued HaH stays were retained according to the corresponding homogeneous group of tariff. The association we retained is: PCM 04 “post-surgical treatment”, ACM 03 or ACM 11 (same weight) “intravenous treatments” or “orthopedic rehabilitation”, Karnofsky index 70-80 “the patient is able to perform normal activities of daily living with effort, some minor symptoms or signs of the disease” or “the patient is able to take care of himself, but is unable to lead a normal life or work”.

This association corresponds to one of the lowest tariff possible for HaH so our estimation is conservative. In sensitivity analysis, the uncertainty surrounding this hypothesis was tested.

**Rehabilitation care**

From patient files, the number of days was collected for each identified stay in a rehabilitation hospital. Patients included in the study stayed in several different rehabilitation hospitals, as such it was not possible to collect pricing information for each patient. Moreover, in France, rehabilitation care funding is calculated based on 10% on activity-based pricing and for 90% on overall endowment. The assumption that rehabilitation care funding is 100% based on activity pricing was made. A daily cost of rehabilitation care was estimated from pricing information collected for each stay (N=311) at 2 hospitals (Hospices Civils de Lyon and at the Val Rosay hospital) and used to calculate rehabilitation care for all the patients included in this study. In the sensitivity analysis we tested the uncertainty surrounding these hypotheses.

**Out-hospital costs**

Outpatient oral and/or intravenous antibiotic treatments, consultations, imaging and biology acts were retained only if they were related to the management of the PJI and if they did not correspond to an episode of in-hospital care, as these costs would be included in the diagnosis related group tariff. Resource consumptions were valued using the current reimbursement tariff of the French health insurance.

Although, there were a substantial number of biology acts for each patient in the database, some of them would have only a negligible impact on the overall result (tariff < 1€). Moreover, it was also difficult to determine which specific biology acts were related to the disease of interest. Therefore, we chose to focus on the five biological checkups that are most frequently used for the management of hip or knee prosthesis: standard biology including complete blood count or hemogram, blood electrolytes, creatinine, glutamic oxaloacetic transaminase, glutamic pyruvic transaminase, alkaline phosphatase, gamma glutamyl transpeptidase, bilirubine and c reactive protein; cytochemistry of joint fluid; bacteriological examination; anatomopathological examination; antibiotic dosage.

These five biological checkups were included in the analysis only when they were not included in a hospital stay and also valued according to the current reimbursement tariff of the French health insurance.
