# Supplementary material for: Economic Study of 2-Stage Exchange in Patients With Knee or Hip Prosthetic Joint Infection Managed in a Referral Center in France: Time to Use Innovative(s) Intervention(s) at the Time of Reimplantation to Reduce the Risk of Superinfection
Source: Front Med (Lausanne). 2021 May 10;8:552669. doi: 10.3389/fmed.2021.552669 (PMC8142816; doi:10.3389/fmed.2021.552669)
Supplement: Supplementary file 2 [file Table_1.docx]

Supplementary Material S.2. Detailed overview of the management of the 18 patients with a failure (new infection).

| **Patient ID** | **Surgical treatment of the failure** | **Time to failure (weeks)** | **Suppressive antibiotic treatment after the treatment failure** | **Other surgery during the 2-years follow-up** |
| --- | --- | --- | --- | --- |
| 1 | DAIR + mobile part exchange | 2 | NO | NO |
| 2 | DAIR + mobile part exchange | 20 | YES | YES, Dislocation |
| 3 | DAIR + mobile part exchange | 5 | NO | NO |
| 4 | DAIR + mobile part exchange | 4 | NO* | NO |
| 5 | DAIR + mobile part exchange | 2 | NO | NO |
| 6 | DAIR | 1 | YES | NO |
| 7 | DAIR + mobile part exchange | 3 | NO | NO |
| 8 | DAIR + mobile part exchange | 6 | NO | NO |
| 9 | DAIR + mobile part exchange | 5 | YES | NO |
| 10 | DAIR + mobilization of the flap | 2 | NO | NO |
| 11 | DAIR + mobile part exchange | 16 | YES | NO |
| 12 | DAIR + mobile part exchange | 104 | NO | YES, Dislocation |
| 13 | DAIR | 61 | YES | NO |
| 14 | Screw removal | 9 | YES | NO |
| 15 | DAIR + mobile part exchange | 31 | YES | NO |
| 16 | DAIR + mobile part exchange | 45 | YES | NO |
| 17 | DAIR + mobile part exchange | 54 | YES | NO |
| 18 | DAIR | 21 | YES | NO |

*death of patient 18 days after the surgery for failure - death related to PJI
